# Supplementary material for: Spatial mapping of b-value and fractal dimension prior to November 8, 2022 Doti Earthquake, Nepal
Source: PLoS One. 2023 Aug 9;18(8):e0289673. doi: 10.1371/journal.pone.0289673 (PMC10411762; doi:10.1371/journal.pone.0289673)
Supplement: Attachment — Submitted filename: Comments.pdf [file pone.0289673.s001.pdf]

“Spatial mapping of b-value and Fractal Dimension Prior to November 8, 2022 Doti Earthquake, Nepal” by Ram Krishna Tiwari and Harihar Paudyal

After the earthquake with a magnitude 5.6 mb in western Nepal on 8 November 2022, the study focuses on calculation of Gutenberg-Richter b-value of earthquake distribution and correlation fractal dimension (D2) for 493 earthquakes with magnitude of completeness 3.6 in western Nepal Himalaya and adjoining area for the period 1964 to 2022 for the analysis. The averaged low b-value and high D2 values show the significance of a high hazard zone in this area for an impending earthquake.

The paper can be published after a few minor corrections and please see the attached file.
