# Supplementary material for: Spatial mapping of b-value and fractal dimension prior to November 8, 2022 Doti Earthquake, Nepal
Source: PLoS One. 2023 Aug 9;18(8):e0289673. doi: 10.1371/journal.pone.0289673 (PMC10411762; doi:10.1371/journal.pone.0289673)

PLOS ONE

Spatial mapping of b-value and Fractal Dimension Prior to November 8, 2022 Doti Earthquake, Nepal

--Manuscript Draft--

**Manuscript Number:**	PONE-D-23-00014
**Article Type:**	Research Article
**Full Title:**	Spatial mapping of b-value and Fractal Dimension Prior to November 8, 2022 Doti Earthquake, Nepal
**Short Title:**	b-value and fractal dimension
**Corresponding Author:**	Ram Krishna Tiwari<br>Central Department of Physics<br>Kathmandu, Nepal, NEPAL
**Keywords:**	Western Nepal; power law; Thrust fault; Moment Tensor
**Abstract:**	An earthquake of magnitude 5.6 mb (6.6 ML) hit western Nepal (Doti region) in the wee hours of Wednesday morning local time (2:12 AM, 2022.11.08) killing at least six people. Gutenberg-Richter b-value of earthquake distribution and correlation fractal dimension (D2) are determined for 493 earthquakes with magnitude of completeness 3.6 prior to this earthquake. We consider earthquakes in western Nepal Himalaya and adjoining region (80.0 – 83.5°E and 27.3 –30.5°N) for the period 1964 to 2022 for the analysis. The b-value 0.68 [[EQUATION]] implies a highly stress zone and the spatial correlation [[EQUATION]] implies a highly heterogeneous region where the epicenters are spatially distributed. Low b-values and high D2 values identifies the study region as a high hazard zone. Focal mechanism styles and low b-values correlate with thrust nature of earthquakes and show that the earthquake's occurrence is associated with the dynamics of the faults responsible for generating the past earthquakes.
**Order of Authors:**	Ram Krishna Tiwari
	Harihar Paudyal
**Opposed Reviewers:**	
**Additional Information:**	
**Question**	**Response**
**Financial Disclosure**<br><br>Enter a financial disclosure statement that describes the sources of funding for the work included in this submission. Review the submission guidelines for detailed requirements. View published research articles from *PLOS ONE* for specific examples.<br><br>This statement is required for submission and **will appear in the published article** if the submission is accepted. Please make sure it is accurate.	no

Powered by Editorial Manager® and ProduXion Manager® from Aries Systems Corporation

**Unfunded studies**<br>Enter: *The author(s) received no specific funding for this work.*<br><br>**Funded studies**<br>Enter a statement with the following details:<br>• Initials of the authors who received each award<br>• Grant numbers awarded to each author<br>• The full name of each funder<br>• URL of each funder website<br>• Did the sponsors or funders play any role in the study design, data collection and analysis, decision to publish, or preparation of the manuscript?<br>• **NO** - Include this sentence at the end of your statement: *The funders had no role in study design, data collection and analysis, decision to publish, or preparation of the manuscript.*<br>• **YES** - Specify the role(s) played.<br><br>* typeset	
**Competing Interests**<br><br>Use the instructions below to enter a competing interest statement for this submission. On behalf of all authors, disclose any competing interests that could be perceived to bias this work—acknowledging all financial support and any other relevant financial or non-financial competing interests.<br><br>This statement is required for submission and **will appear in the published article** if the submission is accepted. Please make sure it is accurate and that any funding sources listed in your Funding Information later in the submission form are also declared in your Financial Disclosure statement.<br><br>View published research articles from *PLOS ONE* for specific examples.	no competing interest

Powered by Editorial Manager® and ProduXion Manager® from Aries Systems Corporation

**NO authors have competing interests**<br><br>Enter: *The authors have declared that no competing interests exist.*<br><br>**Authors with competing interests**<br><br>Enter competing interest details beginning with this statement:<br><br>*I have read the journal's policy and the authors of this manuscript have the following competing interests: [insert competing interests here]*<br><br>* typeset	
**Ethics Statement**<br><br>Enter an ethics statement for this submission. This statement is required if the study involved:<br><br>• Human participants<br>• Human specimens or tissue<br>• Vertebrate animals or cephalopods<br>• Vertebrate embryos or tissues<br>• Field research<br><br>Write "N/A" if the submission does not require an ethics statement.<br><br>General guidance is provided below. Consult the submission guidelines for detailed instructions. **Make sure that all information entered here is included in the Methods section of the manuscript.**	N/A

**Format for specific study types**<br><br>**Human Subject Research (involving human participants and/or tissue)**<br>• Give the name of the institutional review board or ethics committee that approved the study<br>• Include the approval number and/or a statement indicating approval of this research<br>• Indicate the form of consent obtained (written/oral) or the reason that consent was not obtained (e.g. the data were analyzed anonymously)<br><br>**Animal Research (involving vertebrate animals, embryos or tissues)**<br>• Provide the name of the Institutional Animal Care and Use Committee (IACUC) or other relevant ethics board that reviewed the study protocol, and indicate whether they approved this research or granted a formal waiver of ethical approval<br>• Include an approval number if one was obtained<br>• If the study involved *non-human primates*, add *additional details* about animal welfare and steps taken to ameliorate suffering<br>• If anesthesia, euthanasia, or any kind of animal sacrifice is part of the study, include briefly which substances and/or methods were applied<br><br>**Field Research**<br><br>Include the following details if this study involves the collection of plant, animal, or other materials from a natural setting:<br>• Field permit number<br>• Name of the institution or relevant body that granted permission	
**Data Availability**<br><br>Authors are required to make all data underlying the findings described fully available, without restriction, and from the time of publication. PLOS allows rare exceptions to address legal and ethical concerns. See the PLOS Data Policy and FAQ for detailed information.	Yes - all data are fully available without restriction

A Data Availability Statement describing where the data can be found is required at submission. Your answers to this question constitute the Data Availability Statement and **will be published in the article**, if accepted.<br><br>**Important:** Stating 'data available on request from the author' is not sufficient. If your data are only available upon request, select 'No' for the first question and explain your exceptional situation in the text box.<br><br>Do the authors confirm that all data underlying the findings described in their manuscript are fully available without restriction?	
**Describe where the data may be found in full sentences. If you are copying our sample text, replace any instances of XXX with the appropriate details.**<br><br>• If the data are **held or will be held in a public repository**, include URLs, accession numbers or DOIs. If this information will only be available after acceptance, indicate this by ticking the box below. For example: *All XXX files are available from the XXX database (accession number(s) XXX, XXX.*).<br>• If the data are all contained **within the manuscript and/or Supporting Information files**, enter the following: *All relevant data are within the manuscript and its Supporting Information files.*<br>• If neither of these applies but you are able to provide **details of access elsewhere**, with or without limitations, please do so. For example:<br><br>*Data cannot be shared publicly because of [XXX]. Data are available from the XXX Institutional Data Access / Ethics Committee (contact via XXX) for researchers who meet the criteria for access to confidential data.*<br><br>*The data underlying the results presented in the study are available from (include the name of the third party*	free available

and contact information or URL).<br>• This text is appropriate if the data are owned by a third party and authors do not have permission to share the data.<br><br>* typeset	
Additional data availability information:	

Powered by Editorial Manager® and ProduXion Manager® from Aries Systems Corporation

TRIBHUVAN UNIVERSITY

BIRENDRA MULTIPLE CAMPUS

Bharatpur, Chitwan, Nepal

To **Date: 28 December 2022**

The Editor

Plos One

Sir/ Madam,

We are herewith resubmitting a manuscript in word format entitled **"Spatial mapping of b-value and Fractal Dimension Prior to November 8, 2022 Doti Earthquake, Nepal ",** by Ram Krishna Tiwari and Harihar Paudyal for possible publication under the banner of **Plos One**. It is also declared that this work has no conflict of interest. Thank you in advance for your kind cooperation.

Sincerely,

Harihar Paudyal, PhD

Professor

Address for Corresponding Author:

Ram Krishna Tiwari

Central Department of Physics, Tribuvan Univerity, Kirtipur, Kathmandu, Nepal and

Birendra Multiple Campus, Tribhuvan University, Bharatpur, Chitwan, Nepal

E-mail: ram.tiwari@bimc.tu.edu.np

Co-author:

Professor **Harihar Paudyal, PhD**

Department of Physics, Birendra Multiple Campus, Tribhuvan University, Bharatpur, Chitwan, Nepal

E-mail:hariharpaudyal@gmail.com

Contact No.: +977- 985506587

Click here to access/download;Manuscript;RKT_HP_DOTIplosone_MS_20230

Spatial mapping of b-value and Fractal Dimension Prior to November 8, 2022 Doti Earthquake, Nepal

Ram Krishna Tiwari [1, 2] Harihar Paudyal [2]

[1]Central Department of Physics, Tribhuvan University, Kirtipur, Kathmandu, Nepal
[2]Birendra Multiple Campus, Tribhuvan University, Bharatpur, Chitwan, Nepal,

Corresponding email: ram.tiwari@bimc.tu.edu.np

Abstract

An earthquake of magnitude 5.6 mb (6.6 ML) hit western Nepal (Doti region) in the wee hours of Wednesday morning local time (2:12 AM, 2022.11.08) killing at least six people. Gutenberg-Richter b-value of earthquake distribution and correlation fractal dimension (D_2) are determined for 493 earthquakes with magnitude of completeness 3.6 prior to this earthquake. We consider earthquakes in western Nepal Himalaya and adjoining region (80.0 – 83.5°E and 27.3 –30.5°N) for the period 1964 to 2022 for the analysis. The b-value 0.68 $\pm$0.03 implies a highly stress zone and the spatial correlation 1.81 $\pm$ 0.02 implies a highly heterogeneous region where the epicenters are spatially distributed. Low b-values and high D_2 values identifies the study region as a high hazard zone. Focal mechanism styles and low b-values correlate with thrust nature of earthquakes and show that the earthquake's occurrence is associated with the dynamics of the faults responsible for generating the past earthquakes.

Keywords: Western Nepal, power law, Thrust fault, Moment Tensor

Introduction

The magnitude distribution of earthquakes, the spatial distribution of epicenter/hypocenter, frequency of aftershocks etc., satisfy the power law distribution (Pilgrim & Taylor, 2019; Roy et al., 2007). Hence, earthquakes can be described by the scaling parameter obeying power law, known as the fractal dimension (Huang & Turcotte, 1988; Mandlebrot, 1989; Shcherbakov et al., 2005). The b-value of frequency-magnitude is a power law involving magnitude while the two-point spatial correlation dimension of earthquake's epicenter distribution displays a power law that quantifies the proportion of randomness and clusterization. The b-value measures the material heterogeneities within a fault zone and decreases with increasing stress in the brittle part of the earth's crust. It suggests an increase possibility of happening of large magnitude earthquakes (lower b-value) and smaller magnitude earthquakes (higher b-value) (Gulia & Wiemer, 2010; Ogata & Tsuruoka, 2016; Wyss et al., 2004). The mean value of b ranges from about 0.7 to about 1.0 for longer period (Arroyo-Solórzano & Linkimer, 2021; El-Isa, 2018; Ketthong & Pailoplee, 2012). The b-value linked with the dynamics of individual faults is universal whereas the fractal dimension of the fault network can vary depending on geological heterogeneity (Lomnitz- Adler, 1992). Different tectonic processes undergoing inside the earth generally activate the fault systems in asperity zones from where generous size earthquakes nucleate (Legrand, 2002; Roy et al., 2011)

Nepal rests on the boundary of Eurasian plate and Indian plate with major faults parallel to its length, so always vulnerable to earthquakes (Bai et al., 2016; Pei et al., 2016). From northern belt to the southern belt, the South Tibetan Detachment (STD), Main Central Thrust (MCT), Main Boundary Thrust (MBT), and Main Frontal Thrust (MFT) are major faults system in the Himalaya (Grandin et al., 2015; Upreti, 1999). The MCT, MBT, and MFT sole into a gentle dipping detachment called the Main Himalayan Thrust (MHT)

(Sathiakumar & Barbot, 2021). The present-day convergence between the Indian and Eurasian plates is mostly accommodated along the MHT whose surface trace is MFT. Along with these major faults, some northeast-southwest trending transverse lineaments like Tanakpur lineament, Karnali lineament, and Samea lineament are also responsible for seismicity activity of the western Nepal (Paudyal et al., 2010). In the past work related with anomalous seismicity of western Nepal Himalaya, potential zone for the medium size earthquakes was identified within an area bounded by 29.3°-30.5°N and 81.2°-81.9°E (Paudyal & Singh, 2008). The recent work identified the existence of asperity capable of hosting the future earthquake with similar character of the 2015 Gorkha earthquake towards west of the epicenter of Gorkha earthquake (Sreejith et al., 2018). The research carried out by separate groups (Bilham, 2019; Mencin et al., 2016; Molnar & Pandey, 1989) highlights the presence of a crucial seismic gap in western Nepal and adjoining region. Thus, the prime objective of this paper is to characterize the stress level and heterogeneity of the seismogenic sources by mapping b-value and fractal dimension in the region.

In record, Nepal has the long history of moderate to large earthquakes since 1255 (Joshi & Kaushik, 2017). The western region of Nepal is seismically most active segment of central Himalaya and had also hosted many earthquakes in the past and few of them are notable (Paudyal et al., 2010). An earthquake of 26 September, 1964 (6.0 mb) occurred in Nepal- India border 4 km from Dharchula, Uttarakhand, India. The 6.0 mb earthquake on 27 June 1966, at the depth of 23.80 km in border of Nepal and India took the lives of 80 people (Agrawal, 1969). The 6.1 mb Bajhang earthquakes on July 29, 1980 affected Baitadhi, Bajhang and Darchula region of western Nepal and took 125 lives (Chaulagain et al., 2018; Khanal, 1997). Following the history, a magnitude 5.6 mb or 6.6 ML earthquake hit western Nepal in the wee hours of Wednesday morning local time (2:12 AM, 2022.11.08) killing at least six people (Pokharel & Wang, 2022). The epicenter of the quake was 21 km east of Dipayal (Headquarter of Doti District), at a depth of 15.7 km. Cracks have surfaced in most of the houses in the aftermath of the geological event. As per the National Earthquake Monitoring & Research Center (seismonepal.gov.np), a 5.7 ML earthquake was reported in the area at 9:07 pm prior to the stronger one that was felt later. Many aftershocks had been felt after the two bigger quakes throughout the night. The jolt was felt in and around Bajhang, Kailali, Kanchanpur, Banke, Rukum west and far up to the Indian capital Delhi and lasted for about 10 seconds (Joshi, 2022). The fault plane solutions from the catalog of Global Centroid Moment Tensor (GCMT) (Dziewonski et al., 1981; Ekström et al., 2012) depict the Doti earthquake as shallow angle north east dipping thrust fault event. The fault geometry of this earthquake is similar to the 1980, Bajhang earthquake (Figure 1).

Figure 1. Western Nepal and adjoining regions showing moderate historical earthquakes, viz.1964 earthquake, 1966 earthquake, and 1980 Bajhang earthquake (Yellow stars). Recent Doti earthquake (2022) is represented by red star. Black sphere is indicating the Dipayal (Head quarter of Doti district). Tiny spheres (orange) indicate the earthquakes having magnitude between 3.6 and 5.0 and blue spheres indicate the earthquakes having magnitude between 5.1 and 6.0. Beach balls depict the focal mechanism solutions of July 29, 1980 Bajhang earthquake and 8 November, 2022 Doti earthquake. STD is South Tibetan Detachment, MCT is Main Central Thrust, MBT is the Main Boundary Thrust, and MFT is the Main Frontal Thrust. TL is the Tanakpur lineament, KL is Karnali lineament, and SL is Samea lineament (Paudyal et al., 2010).

Data and methodology

For better understanding of earthquake phenomena, a decent dataset is essential. The dataset used in this study is from the catalog of International Seismological Centre (ISC) (Bondár & Storchak, 2011; Storchak et al., 2020). We retrieved 634 earthquakes from the ISC catalog. After declustering and removing dependent events (Reasenberg, 1985), only 609 earthquakes were retained. The completeness magnitude (Mc) is checked by the ZMAP software (Wiemer, 2001) which gives only 493 events with Mc ≥ 3.6 for final analysis (Figure 2 and Figure 3). The final data set contains 442 events having magnitude between 3.6 and 4.9, 48 events having magnitude between 5.0 and 5.9, and three events having magnitude greater than equal to 6.0. To map the b-value and fractal dimension, the study area was gridded at 1° ×1° spacing with an overlapping of 0.2°. The correlation dimension (D_2) and b-value are estimated only for the grids containing events ≥ 25 for the reliable values of these parameters. The b-value is calculated by maximum likelihood estimation (MLE) method which has an advantage of not being influenced by large magnitude earthquakes. The formula for the estimation of b-value (Aki, 1965; Bender, 1983) with standard deviation (Shi & Bolt, 1982) is

$$b = \frac{\log_{10} e}{M_a - \left(M - \frac{\Delta \mathrm{M}}{2}\right)}$$

Where M_a is average magnitude, M is minimum magnitude, and ΔM is binning width of the catalog. The spatial correlation dimension is calculated from correlation integral function $c(r)$ (Mondal et al., 2019; Roy & Mondal, 2009; Roy & Padhi, 2007) as,

$$c(r) = \frac{2}{N(N-1)} \sum_{\substack{i,j=1 \\ i \neq j}}^{N} H(r - |X_i - X_j|)$$

Where r is the scaling radius (r = 5 km to r = 35 km for this study), N is the number earthquakes in the sample grid, $X_i - X_j$ is the angular distance between two events which can be evaluated by the spherical triangle method (Hirata, 1989), and H (.) is the Heaviside step function. The slope of the linear part of plot for $logr - \log c(r)$ gives the correlation fractal dimension (D_2) as depicted for the window containing all 493 earthquakes (Figure 4). The estimated values of D_2 and b are presented in the table 1.

Figure 2. Magnitude histogram of the dataset under study

Figure 3. Magnitude of completeness (Mc) of dataset and b-value of earthquake distribution with coefficient of determination (R^2)

Results and discussion

From the earthquake data of ISC catalog, we have estimated the b-value and fractal dimension of 39 grids following the procedure mentioned in data and methodology section and presented in table 1.

Table 1. List of estimated parameters of 39 grids including number of events in grid, b-value, and fractal dimension (D_2) with standard deviation, and respective coefficient determination (R^2). Grids containing events ≥ 25 are only analyzed for reliable values of estimated parameters.

S.N.	Grid	No. of events	b-value	R^2	D_2	R^2
1	80.00 – 81.00°E<br>27.30 – 28.30°N	0	–	–	–	–
2	80.20 – 81.20°E<br>27.30 – 28.30°N	0	–	–	–	–
3	80.40 – 81.40°E<br>27.30 – 28.30°N	1	–	–	–	–
4	80.60 – 81.60°E<br>27.30 – 28.30°N	2	–	–	–	–
5	80.80 – 81.80°E<br>27.30 – 28.30°N	3	–	–	–	–
6	81.00 – 82.00°E<br>27.30 – 28.30°N	4	–	–	–	–
7	81.20 – 82.20°E<br>27.30 – 28.30°N	4	–	–	–	–
8	81.40 – 82.40°E<br>27.30 – 28.30°N	3	–	–	–	–
9	81.60 – 82.60°E<br>27.30 – 28.30°N	5	–	–	–	–
10	81.80 – 82.80°E<br>27.30 – 28.30°N	5	–	–	–	–
11	82.00 – 83.00°E<br>27.30 – 28.30°N	5	–	–	–	–
12	82.20 – 83.20°E<br>27.30 – 28.30°N	8	–	–	–	–
13	82.40 – 83.50°E<br>27.30 – 28.30°N	9	–	–	–	–
14	80.00 – 81.00°E<br>28.30 – 29.30°N	18	–	–	–	–
15	80.20 – 81.20°E<br>28.30 – 29.30°N	17	–	–	–	–
16	80.40 – 81.40°E<br>28.30 – 29.30°N	28	0.57 ± 0.02	0.89	1.72 ± 0.03	0.99
17	80.60 – 81.60°E<br>28.30 – 29.30°N	47	0.65 ± 0.03	0.86	1.56 ± 0.01	1.00
18	80.80 – 81.80°E<br>28.30 – 29.30°N	62	0.61 ± 0.04	0.88	1.54 ± 0.03	0.99
19	81.00 – 82.00°E<br>28.30 – 29.30°N	68	0.62 ± 0.04	0.88	1.61 ± 0.04	0.99
20	81.20 – 82.20°E<br>28.30 – 29.30°N	72	0.66 ± 0.05	0.85	1.62 ± 0.04	0.99
21	81.40 – 82.40°E	79	0.65 ± 0.05	0.94	1.58 ± 0.04	0.99

	28.30 – 29.30°N					
22	81.60 – 82.60°E<br>28.30 – 29.30°N	63	0.62 ± 0.05	0.96	1.67 ± 0.03	0.99
23	81.80 – 82.80°E<br>28.30 – 29.30°N	50	0.65 ± 0.06	0.95	1.55 ± 0.02	1.00
24	82.00 – 83.00°E<br>28.30 – 29.30°N	49	0.63 ± 0.05	0.97	1.47 ± 0.02	1.00
25	82.20 – 83.20°E<br>28.30 – 29.30°N	42	0.61 ± 0.03	0.96	1.38 ± 0.01	1.00
26	82.40 – 83.50°E<br>28.30 – 29.30°N	34	0.69 ± 0.04	0.92	1.49 ± 0.02	1.00
27	80.00 – 81.00°E<br>29.30 – 30.50°N	133	0.60 ± 0.11	0.88	1.67 ± 0.02	1.00
28	80.20 – 81.20°E<br>29.30 – 30.50°N	147	0.59 ± 0.11	0.87	1.60 ± 0.03	0.99
29	80.40 – 81.40°E<br>29.30 – 30.50°N	140	0.62 ± 0.11	0.84	1.64 ± 0.04	0.99
30	80.60 – 81.60°E<br>29.30 – 30.50°N	146	0.64 ± 0.12	0.83	1.66 ± 0.03	0.99
31	80.80 – 81.80°E<br>29.30 – 30.50°N	171	0.66 ± 0.15	0.82	1.67 ± 0.03	0.99
32	81.00 – 82.00°E<br>29.30 – 30.50°N	159	0.69 ± 0.14	0.81	1.68 ± 0.03	1.00
33	81.20 – 82.20°E<br>29.30 – 30.50°N	141	0.72 ± 0.15	0.84	1.64 ± 0.03	0.99
34	81.40 – 82.40°E<br>29.30 – 30.50°N	120	0.70 ± 0.12	0.86	1.55 ± 0.03	0.99
35	81.60 – 82.60°E<br>29.30 – 30.50°N	83	0.73 ± 0.10	0.81	1.48 ± 0.01	1.00
36	81.80 – 82.80°E<br>29.30 – 30.50°N	40	0.86 ± 0.06	0.76	1.61 ± 0.02	1.00
37	82.00 – 83.00°E<br>29.30 – 30.50°N	36	1.07 ± 0.08	0.72	1.72 ± 0.03	1.00
38	82.20 – 83.20°E<br>29.30 – 30.50°N	25	1.40 ± 0.19	0.72	1.64 ± 0.03	0.99
39	82.40 – 83.50°E<br>29.30 – 30.50°N	30	1.55 ± 0.25	0.67	1.67 ± 0.01	1.00

Figure 4. The method of estimation of correlation fractal dimension as the slope of the linear part of the plot between where r = 5 km to r = 35 km is set as the distance between depopulation and saturation (Nerenberg & Essex, 1990)

Figure 5. b-value contours of the study region. The b-values are calculated for the grids of size 1° ×1° with overlapping of 0.2°. STD, MCT, MBT, MFT, TL, KL, and SL are as mentioned in the captions of the figure 1.

The b-value for whole data set is found to be 0.68 ± 0.03 while the b-value map shows the broad variation for different grids i.e., between 0.48 and 1.55 (Table 1 and Figure 3). This type of variation is acceptable for the seismically active Himalayan region (Gui et al., 2019; Zhu, 2021). The broad patch of low b-value between 0.48 and 0.64 is noticed for the region occupied by historical earthquakes and the recent Doti earthquake as well. These low b-values can also be linked with the reverse faulting mechanism of the region. The lower b-values are indicator of a more stressed zone or presence of asperity in the region (Kumar et al., 2013; Nakaya, 2006). Thus, the area enclosed between Tanakpur Lineament and Samea lineament could be the host region of the future large earthquake. The north east corner of the study region is reflected with high b-value contours (1.00 to 1.50), so can be inferred as less probable region for the future large earthquake.

Figure 6. D-value contours of the study region. The D-values are calculated for the grids of size 1° ×1° with overlapping of 0.2°. STD, MCT, MBT, MFT, TL, KL, and SL are as mentioned in the captions of the figure 1.

Fractal dimension is a measure of resistance of material against the fracture, so the fragile material had a smaller fractal dimension (D_2). The fractal dimension would increase with an increase in the energy density available for fracture (Bardeji, 2018; Turcotte, 1986). D_2 is a measure of spatial clustering and can take a value from 0 to 2. Its value close to 0 signifies that earthquakes are centralized in a small locality and the value close to 2 signifies that the earthquakes are spatially distributed (Kagan, 2007; Mandal & Rodkin, 2011). The fractal dimension value 1.81 ± 0.02 for whole data set (Table 1 and Figure 4) and the variation between 1.36 and 1.92 for different grids indicate that the epicenters are distributed in 2D seismogenic structures. High D_2 contours (1.5 to 1.92) are identified for the area occupied by the past moderate earthquakes and recent Doti earthquake. These areas could be inferred as the asperity zones along the fault systems. The areas east of Dipayal are identified as the low D_2 contours (1.36 – 1.52) area. The higher values of fractal dimensions obtained from this study suggest the presence of spatially distributed heterogeneous faults in the region and the region are seismically active. The smaller b-value zones (0.48 – 0.60) are corroborated with higher D_2 (1.5 – 1.9), implying negative correlation. The areas with low b-value and high D_2 and are expected to accumulate prominent levels of tectonic stress, which could be the risk factor for generating large earthquakes in the future (Mandal et al., 2022; Sarkar et al., 2020; Tiwari & Paudyal, 2021). The area identified as high risk zone in this study is within the zone identified as anomalous seismicity zone in the past work (Paudyal & Singh, 2008).

Conclusion

After the analysis of earthquake dataset for the period of 58 years (1964 – 2022), the b-value earthquake distribution and the fractal dimension (D_2) of epicenter distribution are mapped for the tectonic structures

of western Nepal and adjoining region. The maximum likelihood method is used for the estimation of b-value and correlation integral method is employed for the estimation of fractal dimension. The results identify the region under study as a high hazard zone with low b-values and high D_2 values. A high D_2 value obtained for the region indicates strong heterogeneity at this part of the Himalaya may be due to varied stress level in the crust. The fault geometry of 2022 Doti earthquake and 1980 Bajhang earthquake revealed by focal mechanism solutions show the similar characteristics, so the occurrence of the earthquake could be related to the previous earthquakes. The mapped region can be inferred to be most hazardous, which correlate well with seismic gap mentioned in the past literature. Finally, this study sheds new light in the understanding the characteristics of the seismogenic sources in western Nepal Himalaya.

Acknowledgements

One of the authors (RKT) would like to acknowledge both University Grant Commission (UGC), Nepal and the Tribhuvan University, Nepal for providing PhD fellowship and sabbatical leave, respectively.

Disclosure statement

The author declares no conflict of interests.

References

Agrawal, P. N. (1969). Structural Response Results During The June 27,1966 Earthquake In Nepal-India Border Region. *Bulletin of the Seismological Society of America*, *59*(2), 771–775. https://doi.org/https://doi.org/10.1785/BSSA0590020771

Aki, K. (1965). Maximum likelihood estimate of b in the formula Log N = a - bM and its confidence limits. *Bull Earthq. Res. Inst. Tokyo Univ.*, *43*, 237–239.

Arroyo-Solórzano, M., & Linkimer, L. (2021). Spatial variability of the b-value and seismic potential in Costa Rica. *Tectonophysics*, *814*. https://doi.org/10.1016/j.tecto.2021.228951

Bai, L., Liu, H., Ritsema, J., Mori, J., Zhang, T., Ishikawa, Y., & Li, G. (2016). Faulting structure above the Main Himalayan Thrust as shown by relocated aftershocks of the 2015 Mw7.8 Gorkha, Nepal, earthquake. *Geophysical Research Letters*, *43*(2), 637–642. https://doi.org/10.1002/2015GL066473

Bardeji, S. Z. (2018). *Fractal Distribution of Earthquake Epicenter and Faulting in the Sepidar Anticline , Sw Zagros*. *9*, 20–21.

Bender, B. (1983). Maximum likelihood estimation of b values for magnitude grouped data. *Bulletin of the Seismological Society of America*, *73*(3), 831–851. http://www.bssaonline.org/cgi/content/abstract/73/3/831

Bilham, R. (2019). Himalayan earthquakes: A review of historical seismicity and early 21st century slip potential. *Geological Society Special Publication*, *483*(1), 423–482. https://doi.org/10.1144/SP483.16

Bondár, I., & Storchak, D. (2011). Improved location procedures at the International Seismological Centre. *Geophysical Journal International*, *186*(3), 1220–1244. https://doi.org/10.1111/j.1365-246X.2011.05107.x

Chaulagain, H., Gautam, D., & Rodrigues, H. (2018). Revisiting major historical earthquakes in Nepal: Overview of 1833, 1934, 1980, 1988, 2011, and 2015 seismic events. In *Impacts and Insights of the Gorkha Earthquake*. Elsevier Inc. https://doi.org/10.1016/B978-0-12-812808-4.00001-8

Dziewonski, A. M., Chou, T. A., & Woodhouse, J. H. (1981). Determination of earthquake source parameters from waveform data for studies of global and regional seismicity. *Journal of Geophysical Research*, *86*(B4), 2825–2852. https://doi.org/10.1029/JB086iB04p02825

Ekström, G., Nettles, M., & Dziewoński, A. M. (2012). The global CMT project 2004-2010: Centroid-moment tensors for 13,017 earthquakes. *Physics of the Earth and Planetary Interiors*, *200–201*, 1–9. https://doi.org/10.1016/j.pepi.2012.04.002

El-Isa, Z. H. (2018). Frequency-Magnitude Distribution of Earthquakes. In *Earthquakes - Forecast, Prognosis and Earthquake Resistant Construction*. https://doi.org/10.5772/intechopen.77294

Grandin, R., Vallée, M., Satriano, C., Lacassin, R., Klinger, Y., Simoes, M., & Bollinger, L. (2015). Rupture process of the Mw = 7.9 2015 Gorkha earthquake (Nepal): Insights into Himalayan megathrust segmentation. *Geophysical Research Letters*, *42*(20), 8373–8382. https://doi.org/10.1002/2015GL066044

Gui, Z., Bai, Y., Wang, Z., & Li, T. (2019). Seismic b-value anomalies in the Sumatran region: Seismotectonic implications. *Journal of Asian Earth Sciences*, *173*(August 2018), 29–41. https://doi.org/10.1016/j.jseaes.2019.01.015

Gulia, L., & Wiemer, S. (2010). The influence of tectonic regimes on the earthquake size distribution: A case study for Italy. *Geophysical Research Letters*, *37*(10), 1–6. https://doi.org/10.1029/2010GL043066

Hirata, T. (1989). A correlation between the b value and the fractal dimension of earthquakes. *Journal of Geophysical Research*, *94*(B6), 7507–7514. https://doi.org/10.1029/JB094iB06p07507

Huang, J., & Turcotte, D. L. (1988). Fractal distributions of stress and strength and variations of b-value. *Earth and Planetary Science Letters*, *91*(1–2), 223–230. https://doi.org/10.1016/0012-821X(88)90164-1

Joshi, P. (2022, November 9). Earthquake kills six, injures five others in Doti. *MyRepublica*. https://myrepublica.nagariknetwork.com

Joshi, V. M., & Kaushik, H. B. (2017). Historic earthquake-resilient structures in nepal and other himalayan regions and their seismic restoration. *Earthquake Spectra*, *33*(Special issue 1), S299–S319. https://doi.org/10.1193/121616EQS240M

Kagan, Y. Y. (2007). Earthquake spatial distribution: The correlation dimension. *Geophysical Journal International*, *168*(3), 1175–1194. https://doi.org/10.1111/j.1365-246X.2006.03251.x

Ketthong, T., & Pailoplee, S. (2012). Frequency-magnitude Distribution and Fractal Dimension of the Seismicity along the Sumatra-Andaman Subduction Zone : A Temporal Comparison Bulletin of Earth Sciences of Thailand. *Bulletin of Earth Sciences of Thailand*, *8*, 12–23.

Khanal, K. N. (1997). Source parameters estimation of the 1980 Bajhang Earthquake, far western Nepal. *Journal of Nepal Geological Society*, *15*, 45–51. https://doi.org/10.3126/jngs.v15i0.32123

Kumar, N., Yadav, D. K., Mondal, S. K., & Roy, P. N. S. (2013). Stress drop and its relation to tectonic and structural elements for the meizoseismal region of great 1905 Kangra earthquake of the NW Himalaya. *Natural Hazards*, *69*(3), 2021–2038. https://doi.org/10.1007/s11069-013-0793-9

Legrand, D. (2002). Fractal dimensions of small, intermediate, and large earthquakes. *Bulletin of the Seismological Society of America*, *92*(8), 3318–3320. https://doi.org/10.1785/0120020025

Lomnitz- Adler, J. (1992). Interplay of fault dynamics and fractal dimension in determining Gutenberg & Richter's b- value. *Geophysical Journal International*, *108*(3), 941–944. https://doi.org/10.1111/j.1365-246X.1992.tb03482.x

Mandal, P., & Rodkin, M. V. (2011). Seismic imaging of the 2001 Bhuj Mw7.7 earthquake source zone: B-value, fractal dimension and seismic velocity tomography studies. *Tectonophysics*, *512*(1–4), 1–11. https://doi.org/10.1016/j.tecto.2011.09.004

Mandal, P., Srinagesh, D., Suresh, G., Naresh, B., Singh, D. K., Swathi, K., Devi, A., Raghavan, R. V., Shekar, M., Saha, S., Vengala, P. K., & Gupta, A. K. (2022). Three-dimensional b-value and Fractal Dimension Mapping of the Uttarakhand Himalayan Region. *Journal of Geological Society of India*, *98*, 1365–1379.

Mandlebrot, B. B. (1989). Multifractal Measures, Especially for the Geophysicist. *Pure and Applied Geophysics*, *131*(1/2).

Mencin, D., Bendick, R., Upreti, B. N., Adhikari, D. P., Gajurel, A. P., Bhattarai, R. R., Shrestha, H. R., Bhattarai, T. N., Manandhar, N., Galetzka, J., Knappe, E., Pratt-Sitaula, B., Aoudia, A., & Bilham, R. (2016). Himalayan strain reservoir inferred from limited afterslip following the Gorkha earthquake. *Nature Geoscience*, *9*(7), 533–537. https://doi.org/10.1038/ngeo2734

Molnar, P., & Pandey, M. R. (1989). Rupture zones of great earthquakes in the Himalayan region. *Proceedings of the Indian Academy of Sciences - Earth and Planetary Sciences*, *98*(1), 61–70. https://doi.org/10.1007/BF02880376

Mondal, S. K., Roy, P. N. S., Catherine, J. K., & Pandey, A. K. (2019). Significance of fractal correlation dimension and seismic b-value variation due to 15th July 2009, New Zealand earthquake of mw 7.8. *Annals of Geophysics*, *62*(July 2009), 1–17. https://doi.org/10.4401/ag-8020

Nakaya, S. (2006). Spatiotemporal variation in b value within the subducting slab prior to the 2003 Tokachi-oki earthquake (M 8.0), Japan. *Journal of Geophysical Research: Solid Earth*, *111*(3), 3311. https://doi.org/10.1029/2005JB003658

Nerenberg, M. A. H., & Essex, C. (1990). Correlation dimension and systematic geometric effects. *Physical Review A*, *42*(12), 7065–7074.

Ogata, Y., & Tsuruoka, H. (2016). Statistical monitoring of aftershock sequences: A case study of the 2015 Mw7.8 Gorkha, Nepal, earthquake the 2015 Gorkha, Nepal, Earthquake and Himalayan Studies: First Results 4. Seismology. *Earth, Planets and Space*, *68*(1), 44. https://doi.org/10.1186/s40623-016-0410-8

Paudyal, H., & Singh, H. N. (2008). *Anomalous Seismicity Prceeding Moderate Size Earthquake in Nepal Himalaya and its Adjoining region* (pp. 86–129). Department of Geophysics, Banaras Hindu University,Varanasi, India.

Paudyal, H., Shanker, D., Singh, H., Panthi, A., Kumar, A., & Singh, V. (2010). Current understanding of the seismotectonics of Western Nepal Himalaya and vicinity. *Acta Geodaetica et Geophysica Hungarica*, *45*(2), 195–209. https://doi.org/10.1556/AGeod.45.2010.2.5

Pei, S., Liu, H., Bai, L., Liu, Y., & Sun, Q. (2016). High-resolution seismic tomography of the 2015 Mw7.8 Gorkha earthquake, Nepal: Evidence for the crustal tearing of the Himalayan rift.

Geophysical Research Letters, *43*(17), 9045–9052. https://doi.org/10.1002/2016GL069808

Pilgrim, I., & P. Taylor, R. (2019). Fractal Analysis of Time-Series Data Sets: Methods and Challenges. In *Fractal Analysis*. IntechOpen. https://doi.org/10.5772/intechopen.81958

Pokharel, S., & Wang, P. (2022, November 9). Western Nepal hit by magnitude 5.6 earthquake killing at least six people, officials say. *Cable News Network*. https://www.cnn.com/2022/11/08/asia/delhi-nepal-earthquake-today-nepal-earthquake-today/index.html

Reasenberg, P. (1985). Second-order moment of central California seismicity, 1969-1982. *Journal of Geophysical Research: Solid Earth*, *90*(B7), 5479–5495. https://doi.org/10.1029/jb090ib07p05479

Roy, A., Perfect, E., Dunne, W. M., & McKay, L. D. (2007). Fractal characterization of fracture networks: An improved box-counting technique. *Journal of Geophysical Research: Solid Earth*, *112*(12), 1–9. https://doi.org/10.1029/2006JB004582

Roy, P., & Mondal, S. (2009). Fractal nature of earthquake occurrence in northwest Himalayan region. *J. Indian Geophys. Union*, *13*(2), 63–68.

Roy, P. N. S., & Padhi, A. (2007). Multifractal analysis of earthquakes in the Southeastern Iran-Bam Region. *Pure and Applied Geophysics*, *164*(11), 2271–2290. https://doi.org/10.1007/s00024-007-0272-x

Roy, S., Ghosh, U., Hazra, S., & Kayal, J. R. (2011). Fractal dimension and b-value mapping in the Andaman-Sumatra subduction zone. *Natural Hazards*, *57*(1), 27–37. https://doi.org/10.1007/s11069-010-9667-6

Sarkar, P., Roy, P. N. S., & Pal, S. K. (2020). Rejuvenation of 'pop-up' tectonics for Shillong Plateau in NE Himalayan region. *Journal of Earth System Science*, *129*(1). https://doi.org/10.1007/s12040-020-01389-x

Sathiakumar, S., & Barbot, S. (2021). The stop-start control of seismicity by fault bends along the Main Himalayan Thrust. *Communications Earth & Environment*, *2*(1). https://doi.org/10.1038/s43247-021-00153-3

Shcherbakov, R., Yakovlev, G., Turcotte, D. L., & Rundle, J. B. (2005). Model for the distribution of aftershock interoccurrence times. *Physical Review Letters*, *95*(21). https://doi.org/10.1103/PhysRevLett.95.218501

Shi, Y., & Bolt, A. B. (1982). The standard error of the magnitude-frequency b value. *Bulletin of the Seismological Society of Americaletin*, *72*(5), 1677–1687.

Sreejith, K. M., Sunil, P. S., Agrawal, R., Saji, A. P., Rajawat, A. S., & Ramesh, D. S. (2018). Audit of stored strain energy and extent of future earthquake rupture in central Himalaya. *Scientific Reports*, *8*(1), 1–9. https://doi.org/10.1038/s41598-018-35025-y

Storchak, D. A., Harris, J., Brown, L., Lieser, K., Shumba, B., & Di Giacomo, D. (2020). Rebuild of the Bulletin of the International Seismological Centre (ISC)—part 2: 1980–2010. In *Geoscience Letters* (Vol. 7, Issue 1, pp. 1980–2010). https://doi.org/10.1186/s40562-020-00164-6

Tiwari, R. krishna, & Paudyal, H. (2021). Statistics of the earthquakes in the central Himalaya and its vicinity in last 56 years , with an emphasis in the 25 April 2015 Gorkha , Nepal earthquake. *Contributions to Geophysics and Geodesy*, *51*(4), 321–343. https://doi.org/10.31577/congeo.2021.51.4.2

Turcotte, L. (1986). Fractal and fragmentation. *New York*, *91*(B2), 1921–1926.

Upreti, B. N. (1999). An overview of the stratigraphy and tectonics of the Nepal Himalaya. *Journal of Asian Earth Sciences*, *17*(5–6), 577–606. https://doi.org/10.1016/S1367-9120(99)00047-4

Wiemer. (2001). A software package to analyze seismicity: ZMAP. *Seismological Research Letters*, *72*(3), 373–382. https://doi.org/10.1785/gssrl.72.3.373

Wyss, M., Sammis, C. G., Nadeau, R. M., & Wiemer, S. (2004). Fractal dimension and b-value on creeping and locked patches of the San Andreas fault near Parkfield, California. *Bulletin of the Seismological Society of America*, *94*(2), 410–421. https://doi.org/10.1785/0120030054

Zhu, S. (2021). Estimation of seismic hazard around the Ordos Block of China based on spatial and temporal variations of b-values. *Geomatics, Natural Hazards and Risk*, *12*(1), 2048–2069. https://doi.org/10.1080/19475705.2021.1949394

Click here to access/download;Figure;fig1.p

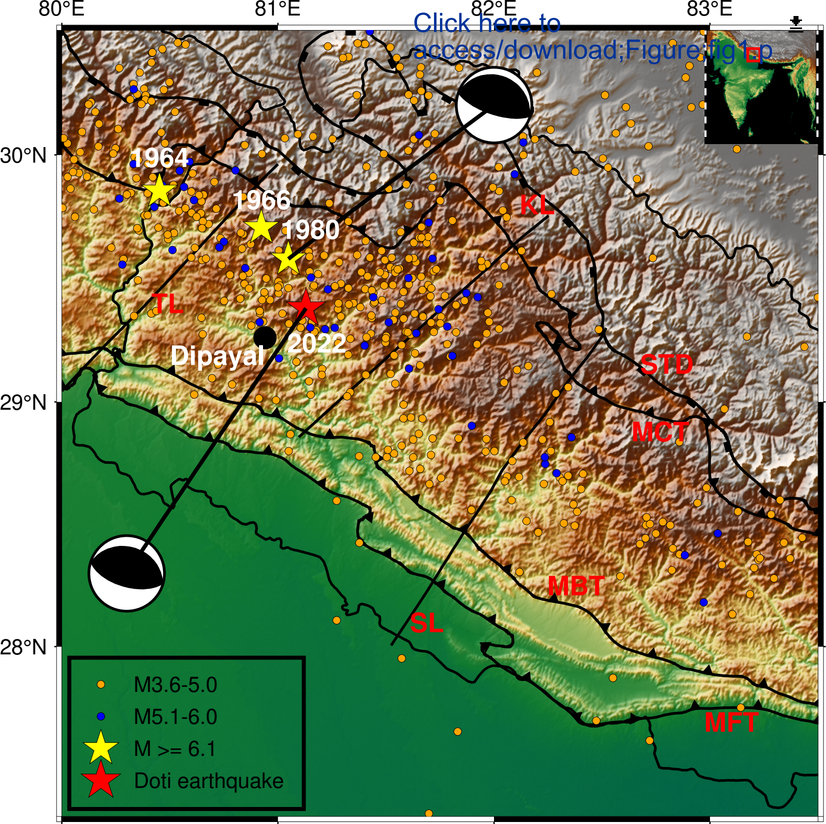

Click here to access/download;Figure;fig2.pdf

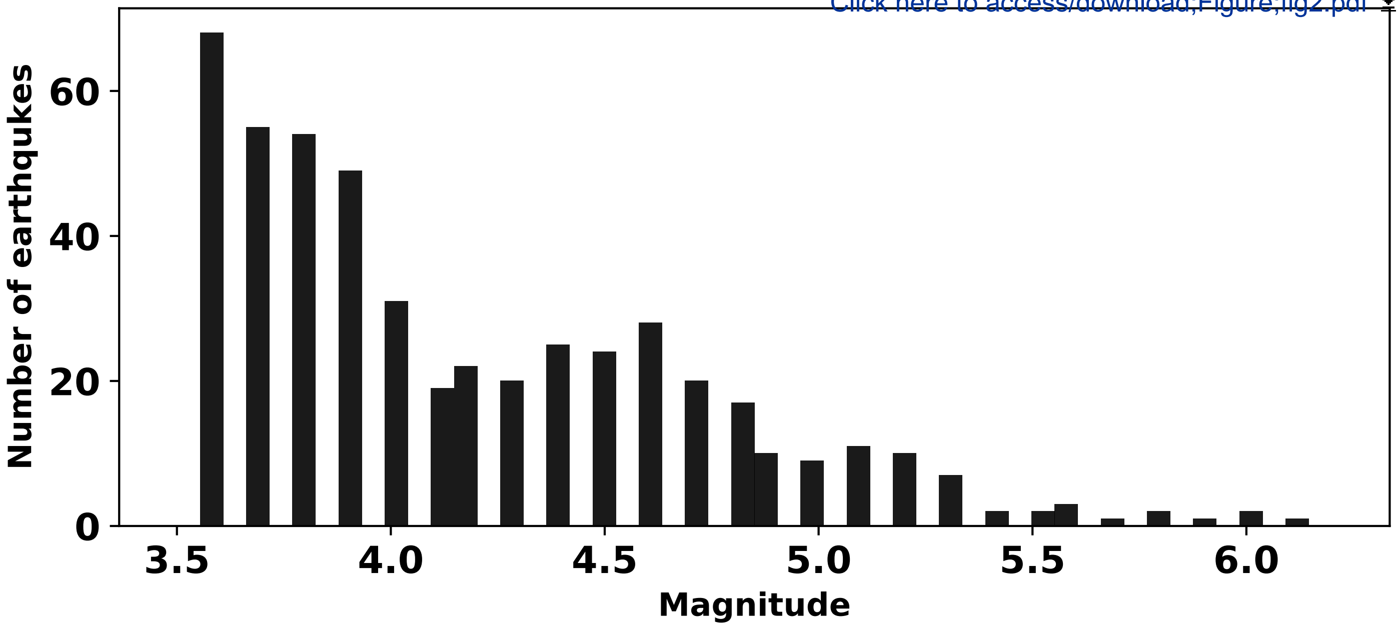

Click here to access/download;Figure;fig3.pdf

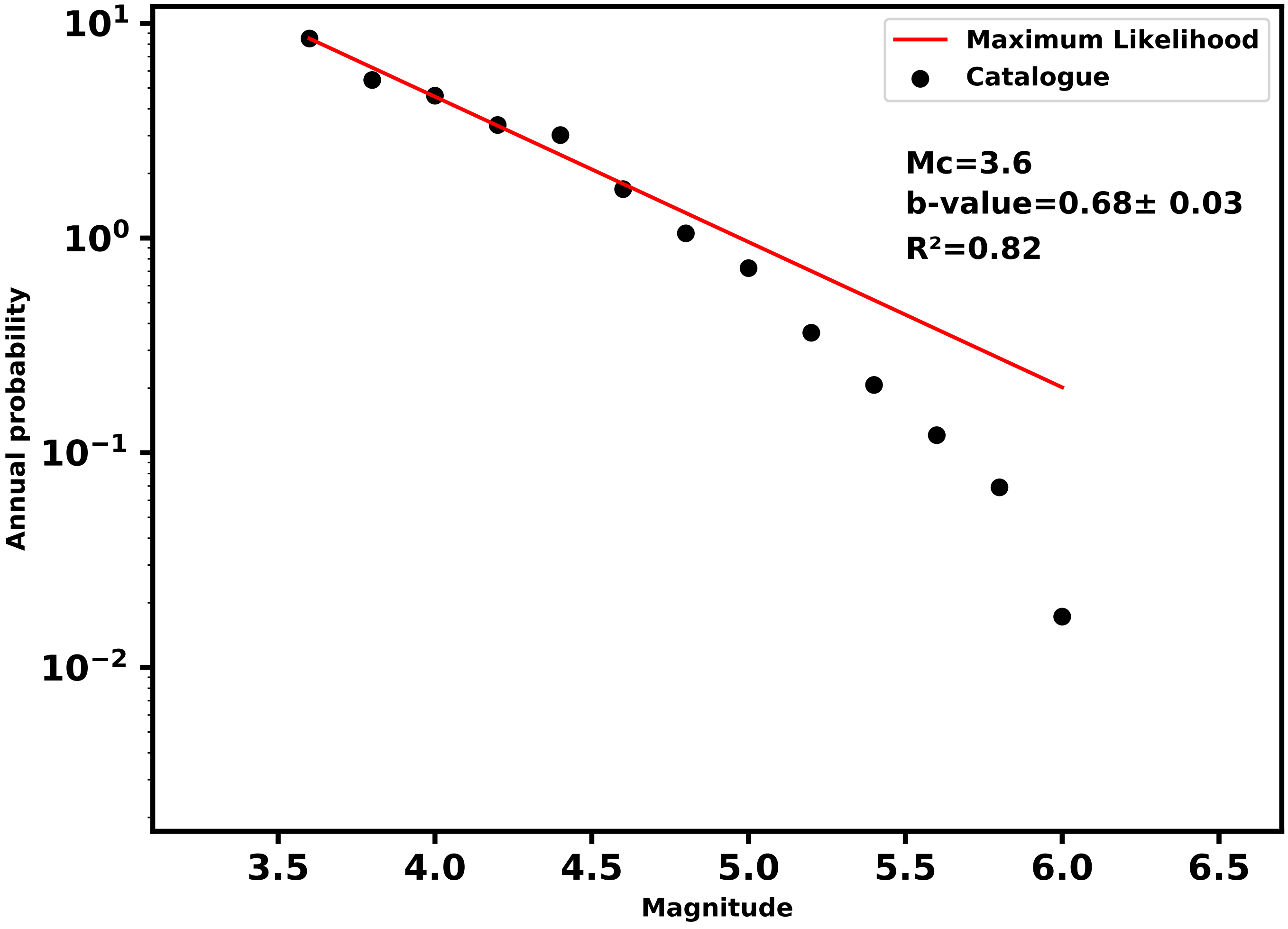

Click here to access/download;Figure;fig4.pdf

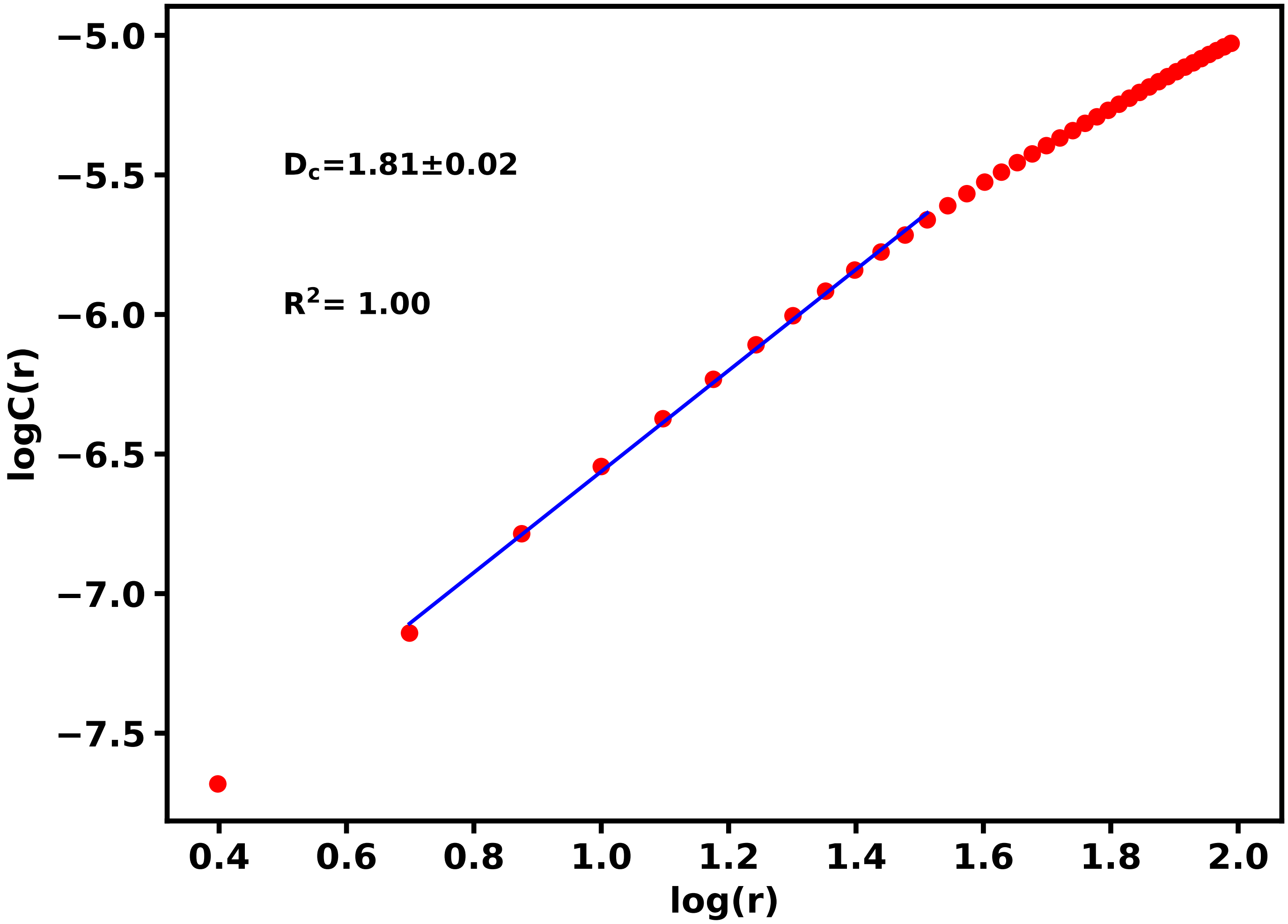

Click here to access/download;Figure;fig5.pdf

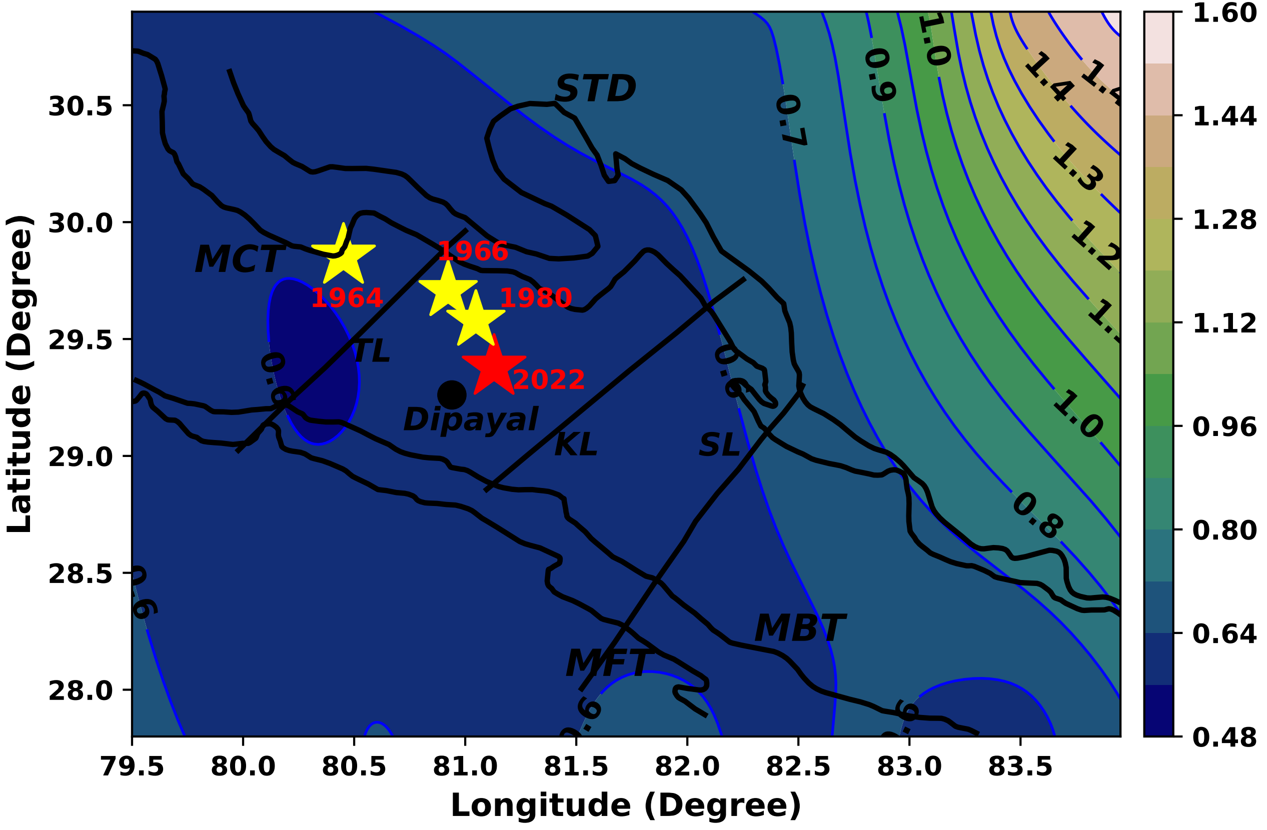

Click here to access/download;Figure;fig6.pdf

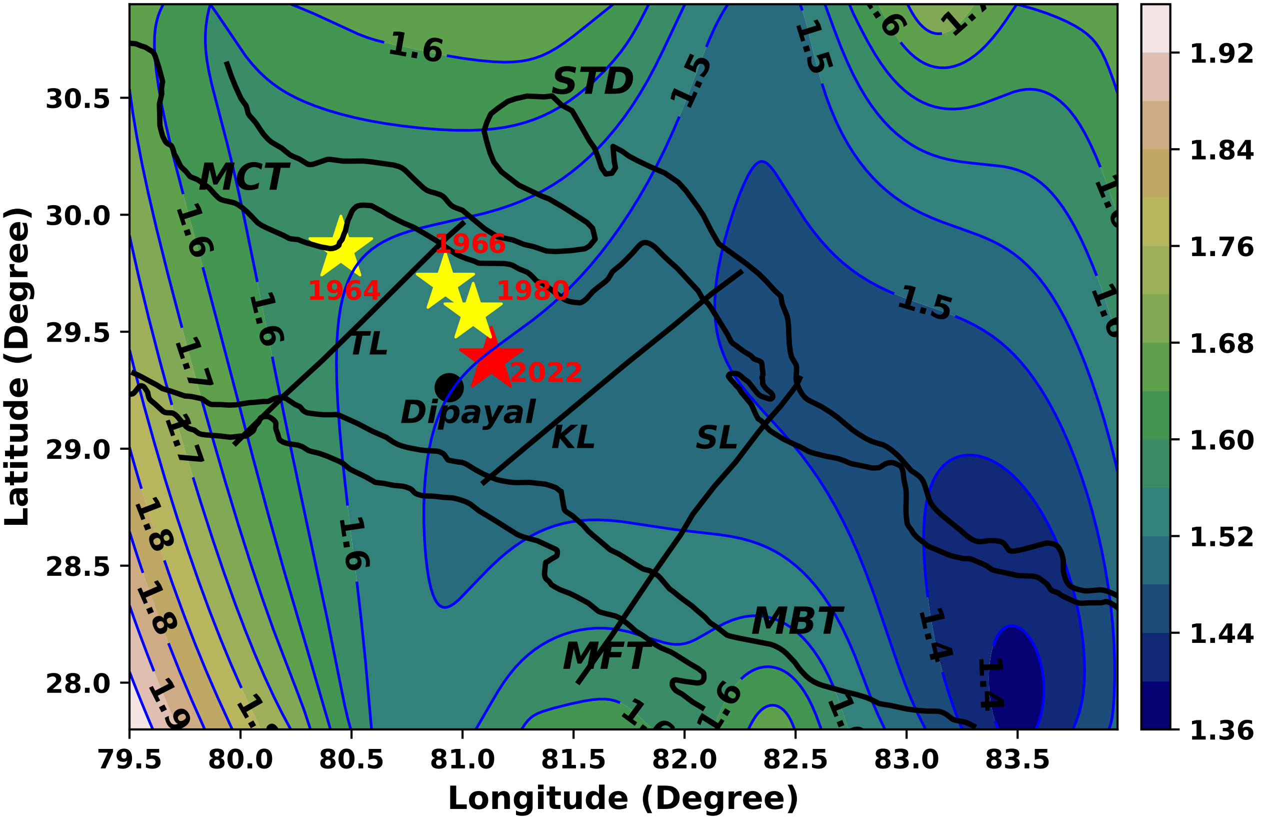

Supplement: Attachment — Submitted filename: PONE-D-23-00014-AE.pdf [file pone.0289673.s003.pdf]
