# Supplementary material for: Spatial mapping of b-value and fractal dimension prior to November 8, 2022 Doti Earthquake, Nepal
Source: PLoS One. 2023 Aug 9;18(8):e0289673. doi: 10.1371/journal.pone.0289673 (PMC10411762; doi:10.1371/journal.pone.0289673)
Supplement: Attachment — Submitted filename: Reviewers_Comments_Response.docx [file pone.0289673.s004.docx]

| SN | Reviewer’s comment | Author’s Response |
| --- | --- | --- |
| 1. | Different tectonic processes undergoing inside the earth generally activate the fault systems in asperity zones from where generous size earthquakes nucleate (Legrand, 2002; Roy et al., 2011)  Reviewer’s suggest to add period in this paragraph | The period is added as decades or centuries |
| 2. | The epicenter of the shock was 21 km east of Dipayal (Headquarter of Doti District), | Quake is replaced by shock |
| 3. | It is better to separate results, discussion and conclusion |  |
| 4. | Please explain why two R^2^ | One is for b-value and other for D_2_ value |
| 5. | Pilgrim, I., & Taylor, R. P. (2019). Fractal Analysis of Time-Series Data Sets: | Now this reference is corrected in the manuscript |
| 6. | for the period should be corrected | It is corrected as for the period of |
| 7. | The b-value 0.68$\pm0.03$ should not be space between 0.68 and plus minus symbol | Spacing is removed from the sentence |
| 8. | A highly stress zone should be high stress zone | It is written as a high stress zone |
| 9 | Identifies the study should be identify | It is now corrected. |
| 10. | Brittle part of the earth’s crust Upper case E | It is now corrected. |
| 11. | (Lomnitz‐ Adler, 1992). | Space is deleted |
| 12. | an increase possibility of happening of | corrected |
| 13. | parallel to its length, so always | corrected |
| 14. | From northern belt to | Corrected as the northern |
| 15. | Responsible for seismicity activity of | Corrected as seismic activity |
| 16. | Magnitude greater than equal to | Corrected as magnitude greater than or equal to |
| 17. | Where Ma is average magnitude, | Where is corrected as where |
| 18. | In Figure 2, why do the bars at 4.1, 4.8, 5.5 have no spacing? | There are earthquakes in cluster with nearly same magnitude |
| 19. | For Figure 5 and 6, the authors may want to add text labels to the color bars to indicate b-value and D2. | Labeling of color bar is now added |
| 20 | The last paragraph of the introduction section is just an enumeration of past events, which is not closely related to the manuscript's main subject. | This paragraph explains the recent seismic activity of the region and we try to correlate recent activity with past large events. This motivates us to prepare the manuscript. |
| 21 | The equations in the data and methodology section are standard. The authors only need to give citations about them and describe how they are used in this study. Showing those equations and the explain them in detail in the manuscript dilutes the substance of the author's own work. | Now we have corrected this portion of the manuscript |
| 22 | How to understand the low correlation between b-value and fractal dimension in Figure 5 and 6? It will be helpful to add seismicity in these two figures too. | Seismicity is added in respective images |
| 23 | The authors briefly touch on focal mechanism. It could be helpful to add more discussion about the relationship between focal mechanism and the calculated b-value and fractal dimension. | This is now mentioned in conclusion section |
| 24 | As the authors plot in Figure 1, there are several large earthquakes in the study region. Are the conclusion for the recent 2022 earthquake also valid for other historical earthquakes? It will be interesting to show how the b-value and fractal dimension change over time. | Now temporal variation of b-value and fractal dimension is added in manuscript |
